# A Discontinuous RNA Platform Mediates RNA Virus Replication: Building an Integrated Model for RNA–based Regulation of Viral Processes

**DOI:** 10.1371/journal.ppat.1000323

**Published:** 2009-03-06

**Authors:** Baodong Wu, Judit Pogany, Hong Na, Beth L. Nicholson, Peter D. Nagy, K. Andrew White

**Affiliations:** 1 Department of Biology, York University, Toronto, Ontario, Canada; 2 Department of Plant Pathology, University of Kentucky, Lexington, Kentucky, United States of America; University of California Riverside, United States of America

## Abstract

Plus-strand RNA viruses contain RNA elements within their genomes that mediate a variety of fundamental viral processes. The traditional view of these elements is that of local RNA structures. This perspective, however, is changing due to increasing discoveries of functional viral RNA elements that are formed by long-range RNA–RNA interactions, often spanning thousands of nucleotides. The plus-strand RNA genomes of tombusviruses exemplify this concept by possessing different long-range RNA–RNA interactions that regulate both viral translation and transcription. Here we report that a third fundamental tombusvirus process, viral genome replication, requires a long-range RNA–based interaction spanning ∼3000 nts. In vivo and in vitro analyses suggest that the discontinuous RNA platform formed by the interaction facilitates efficient assembly of the viral RNA replicase. This finding has allowed us to build an integrated model for the role of global RNA structure in regulating the reproduction of a eukaryotic RNA virus, and the insights gained have extended our understanding of the multifunctional nature of viral RNA genomes.

## Author Summary

Plus-strand (*i.e.* messenger-sensed) RNA viruses are responsible for significant diseases in plants and animals. The single-stranded RNA genomes of these viruses serve as templates for translation of viral proteins and perform other essential functions that generally involve local RNA structures, such as RNA hairpins. Interestingly, plant tombusviruses utilize a number of long-range intra-genomic RNA–RNA interactions to regulate important events during infection of their hosts, *i.e.* viral translation and transcription. Here, we report that an additional essential tombusvirus process, viral RNA replication, also requires a long-range RNA–RNA interaction. Our analyses indicate a role for this RNA–based interaction in the assembly of the viral replicase, which is responsible for executing viral RNA synthesis. This information was used to generate a comprehensive higher-order RNA structural model for functional long-range interactions in the genome of this eukaryotic RNA virus. The model highlights a critical role for global RNA structure in multiple viral processes that are necessary for successful infection of hosts.

## Introduction

Plus-strand RNA viruses infect a wide variety of organisms and are responsible for causing significant diseases in plants, animals, and humans. A key step in the reproduction of these pathogens is replication of their single-stranded RNA genomes. This process occurs in the cytosol of host cells in association with membranes and requires a virally-encoded RNA-dependent RNA polymerase (RdRp) [1]. During infections, the RdRp associates with other viral and host proteins to form the RNA replicase, which is the complex responsible for synthesizing negative-strand RNA intermediates and progeny viral genomes [2]. The mechanism by which replicase assembly occurs is fundamental to understanding genome replication and is currently the focus of intense study [3].

Besides being templates for replication, plus-strand RNA genomes also serve additional functions during infections, including acting as templates for (i) translation of viral proteins, (ii) transcription of viral mRNAs, and (iii) assembly of virus particles. Accordingly, these RNA genomes are multifunctional molecules that possess regulatory mechanisms to ensure that these different processes occur accurately and at the proper time during the infectious cycle. Integral to this control is the presence of different regulatory RNA elements within viral genomes that act as signals for modulating molecular events. Traditionally, such RNA elements have been viewed as localized sequences or structures (*e.g.* RNA hairpins); however, this perspective is rapidly changing due to increasing discoveries of functional viral RNA elements that are formed by long-range RNA–RNA interactions spanning significant distances [4]–[9]. Consequently, our structural concept of a functional viral RNA genome is shifting from that of a “linear” molecule to one that is three-dimensional [4].

**Figure 1:**
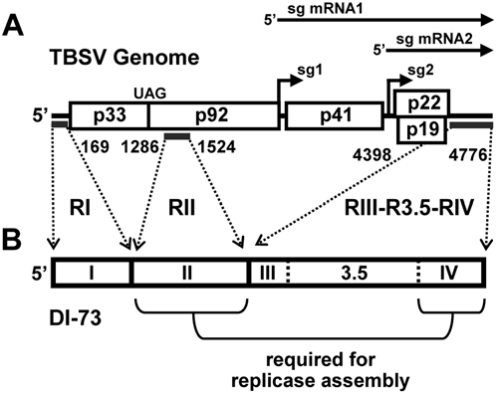
General structure of the TBSV genome and the DI-73 replicon. (A) Schematic linear representation of the TBSV RNA genome with boxes representing encoded proteins. p33 and p92 share a start codon, and both are translated directly from the viral genome; the latter by read-through of the p33 stop codon (UAG). Proteins encoded further downstream are translated from two sg mRNAs that are transcribed during infections (represented as horizontal arrows, top). Regions in the TBSV genome that are present in DI-73 are delineated by thick horizontal lines under the genome. (B) Linear structure of the non-coding DI-73 RNA replicon. The three contiguous regions of DI-73 that are derived from the TBSV genome are delineated by the dotted arrows with the corresponding genomic coordinates. The contiguous 3′-proximal segment is defined by three regions: RIII, R3.5, and RIV. Replicase complex assembly requires both RII and RIV, as indicated.

Tombusviruses, such as Tomato bushy stunt virus (TBSV), have been excellent model systems for understanding molecular aspects of virus reproduction and, particularly, the role of both local and long-range RNA elements in regulating and coordinating the multiple processes that occur during viral infections [10]. The plus-strand RNA genome of TBSV is ∼4.8 kb long and encodes five functional proteins [11]. The RNA replication-related proteins, p33 and its read-through product p92, are translated directly from the genome, while 3′-proximally encoded open reading frames (ORFs) are translated from two subgenomic (sg) mRNAs that are transcribed during infections (Figure 1A) [10]. Interestingly, translation of p33 and p92 occurs via a 5′ cap- and 3′ poly(A) tail-independent mechanism that involves a long-distance RNA–RNA interaction between a 3′ cap-independent translational enhancer (3′CITE) in the 3′-untranslated region (UTR) and the 5′UTR of the genome [12],[13]. Sg mRNA transcription also requires long-range RNA–based interactions within the TBSV genome that involve sequences immediately upstream from transcriptional start sites and partner sequences far upstream [14]–[16]. Accordingly, TBSV utilizes RNA–RNA interactions spanning thousands of nucleotides in two different essential processes: translation and transcription.

**Figure 2:**
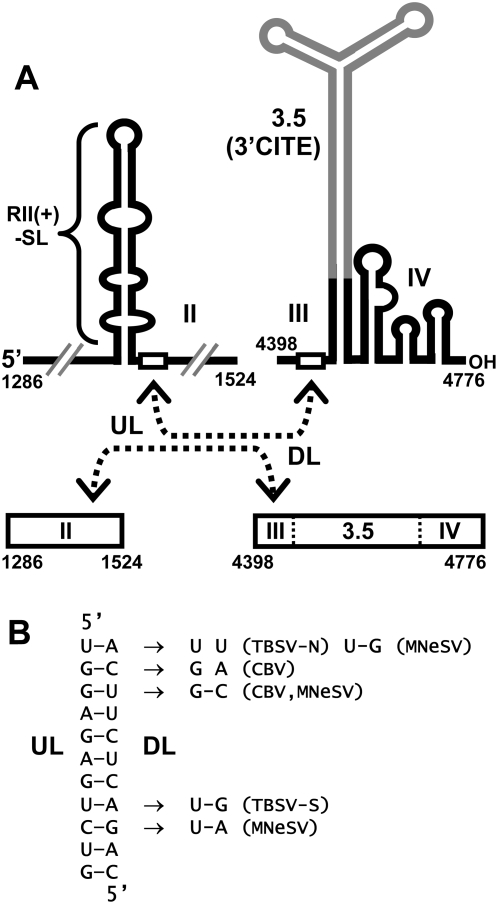
RNA segments of the TBSV genome containing a putative long-range RNA–RNA interaction. (A) Cartoon depicting secondary structures of RII and contiguous segment RIII-R3.5-RIV. RIII and RIV are separated by R3.5 (grey), which forms an extended Y-shaped domain containing the 3′CITE. The relative positions of two complementary 11 nt long sequences in RII and RIII (open rectangles), termed UL and DL, respectively, are shown. The proposed interaction between these two segments in the folded and linear forms of the sequences is indicated by the dashed double-headed arrows. TBSV genome coordinates for the termini of the segments shown are indicated. (B) The putative UL–DL base-pairing interaction between RII and RIII is presented in detail for TBSV (left) and mono- and co-variations that occur in the corresponding sequences in different tombusviruses are shown to the right (TBSV-N, TBSV nipplefruit isolate; TBSV-S, TBSV statice isolate; MNeSV, Maize necrotic streak virus; CBV, Cucumber Bulgarian virus).

Viral RNA replication in TBSV has been studied extensively in both plant and yeast cells and the latter system has served as a genetically-tractable surrogate host that supports authentic viral RNA replication [2],[10],[17]. Two TBSV proteins are required for viral RNA replication: p92, the RdRp, and p33, an auxiliary protein that plays multiple critical roles [10],[18]. Both of these viral proteins are part of the RNA replicase, and several host proteins have also been determined to be components of this complex [19]–[21]. p33 contains peroxisomal targeting signals and transmembrane segments in its N-terminus [22]. This protein also binds, as a dimer, to an internal RNA element in the TBSV genome termed region II (RII; Figure 1) and recruits the genome to peroxisomal membranes, where active RNA replicase is formed [23],[24]. Only the central portion of RII, an extended stem-loop (SL) structure called RII(+)-SL, is required for p33-dimer binding (Figure 2A). p33 also interacts with p92, thus p92 is recruited into replicase assembly by associating with the p33 dimer bound to RII [25]. Interestingly, in addition to the internally-located RII segment, replicase assembly also requires a 3′-terminal section of the viral genome, termed RIV (Figure 1) [26]. RIV is composed of a series of three RNA SLs, which form a compact structure that is located immediately downstream of the 3′CITE (Figure 2A) [27],[28]. Consequently, two non-contiguous RNA elements in the TBSV genome (RII and RIV), separated by ∼3,000 nts, are required for viral replicase assembly [26].

In this report we have pursued the hypothesis that the distantly located RII and RIV require some form of communication to facilitate replicase assembly. Our results support this theory by identifying a long-range RNA–RNA interaction in the TBSV genome that unites RII and RIV and is essential for efficient replicase assembly and viral RNA replication. This finding, along with previous results, has allowed us to generate an integrated higher-order RNA structural model for functional long-range interactions in the genome of a eukaryotic RNA virus. Mechanistic and evolutionary insights provided by this model are discussed.

## Results

### Identification of a potential long-range RNA–RNA interaction in the TBSV genome

Based on the demonstrated requirement for both RII and RIV for replicase assembly in vivo [26], we hypothesized that these two discontinuous RNA elements need to communicate with each other in order to coordinate this event. Considering the abundance of existing long-range RNA–RNA interactions in other fundamental tombusvirus processes [4], we entertained the possibility that the RII-RIV communication might also be RNA-mediated. To this end, all sequenced tombusvirus genomes were analyzed by the RNA secondary structure-predicting program mfold [29],[30] in an attempt to identify candidate RNA–RNA interactions. This analysis revealed a potential RNA base pairing interaction involving two 11 nt long sequences, one located in RII and the other in RIII. The sequence in RII was located just 3′ to the essential RII(+)-SL core structure and was termed upstream linker (UL), while its complementary partner sequence in RIII was termed the downstream linker (DL) (Figure 2A). Although the DL in RIII is located some 267 nts away from RIV in the linear RNA sequence, formation of the intervening and experimentally-confirmed Y-shaped domain (*i.e.* R3.5, which includes the 3′CITE) would position the putative interaction close to RIV in the higher-order RNA structure (Figure 2A) [12],[13],[28]. Thus, the UL–DL interaction would situate RII(+)-SL immediately adjacent to RIV, thereby allowing for communication between these two distal RNA elements. It should be noted that the UL–DL interaction is not a direct interaction between RII and RIV; instead, it could function as an RNA–based bridge that juxtaposes the two RNA elements. The functional relevance of the proposed UL–DL interaction was further supported by comparative sequence analysis of tombusvirus genomes that revealed mono- and co-variations in the two sequences that would either maintain or not significantly disrupt the base pairing interaction (Figure 2B). Accordingly, both thermodynamically-based analysis of secondary structures of full-length viral genomes and comparative sequence analysis of the proposed paired segments support the formation of the UL–DL interaction.

### The UL–DL interaction facilitates viral RNA accumulation in vivo

**Figure 3:**
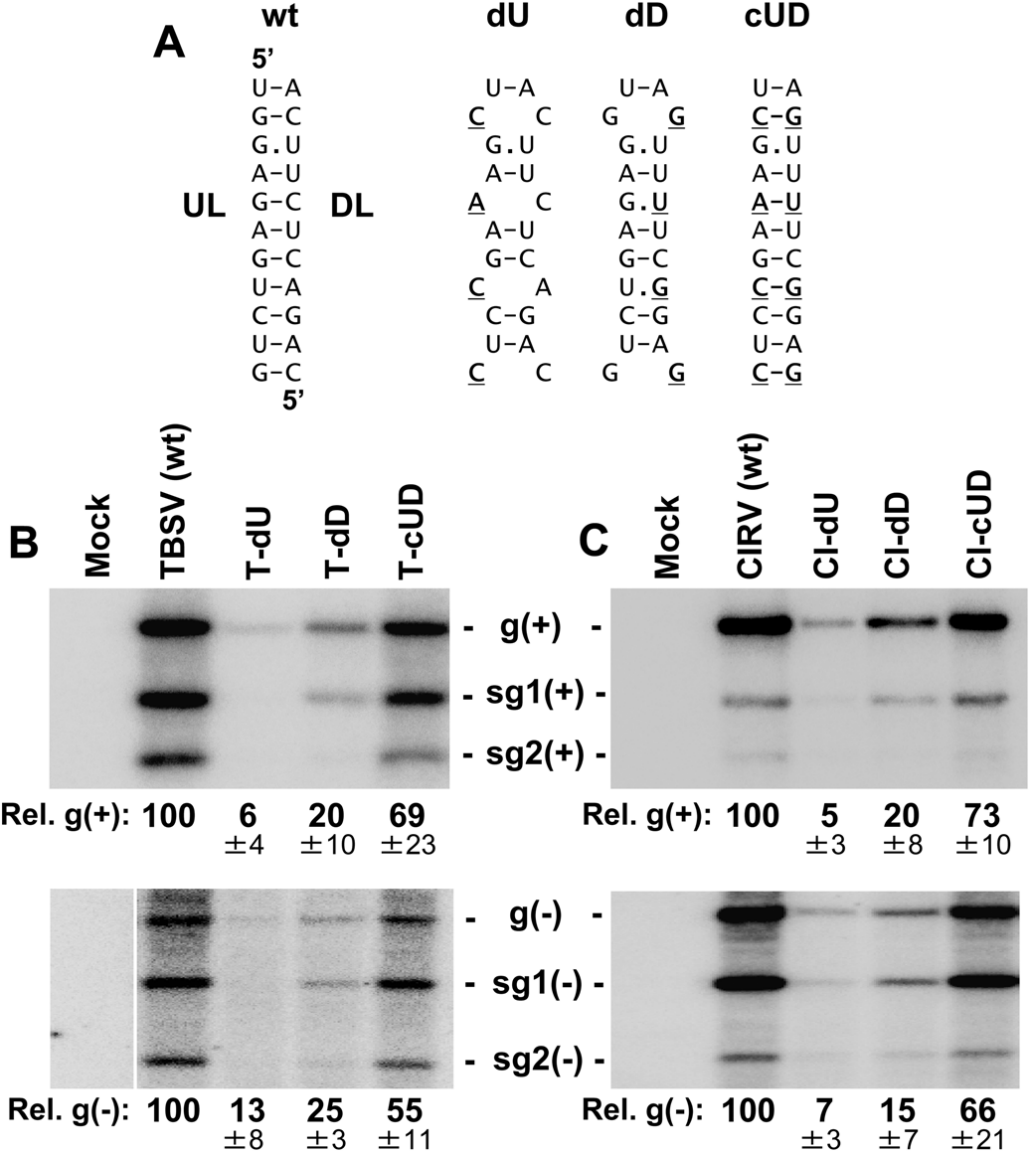
Analysis of the UL–DL interaction in two different tombusvirus genomes. (A) The set of compensatory mutations in the UL and DL sequences that were introduced into the TBSV and CIRV viral genomes is shown. Substituted nucleotides are in bold and underlined. Note that the mutations in dU are predicted to preferentially inhibit the base pairing interaction in the plus-strand (*i.e.* the AC and CC mismatches in the plus-strand would be less disruptive GU wobbles and GG mismatches in the minus strand). Conversely, the dD mutations would favor plus-strand formation (for reasons similar to those described above). (B, C) Northern blot analysis and quantification of plus- and minus-strand accumulation of viral RNAs (top and bottom panels, respectively) from TBSV (B) or CIRV (C) infections of plant protoplasts. The viral genomes analyzed are labeled above the lanes. The positions of the genomes (g) and corresponding subgenomic mRNAs (sg1 and sg2) are indicated. Viral RNAs were analyzed by northern blot analysis 22 hr post-transfection of plant protoplasts. The relative values below the lanes correspond to means (±standard deviations, SD) from three independent experiments and were normalized to the accumulation level of the wt genome, set at 100.

To assess whether the UL–DL interaction was functionally relevant to viral infections, the partner sequences were subjected to compensatory mutational analysis in the context of the TBSV genome. In the genomic mutant T-dU, substitutions were introduced into the UL at wobble positions within the p92 ORF so as not to alter the amino acid sequence of the encoded p92 protein. The changes introduced into T-dU, which were predicted to destabilize the interaction (Figure 3A), reduced genome accumulation levels in plant protoplast infections to ∼6% that of wt TBSV (Figure 3B, top panel). Similarly, disruptive substitutions in the DL in mutant T-dD reduced genome accumulation levels to ∼20% that of wt TBSV (Figure 3B). However, when the two sets of disruptive substitutions were combined in mutant T-cUD, so as to restore base pairing potential, genome accumulation levels showed recovery to ∼69% that of wt TBSV (Figure 3B). These results indicate that the UL–DL interaction is functionally important for efficient TBSV genome accumulation, as well as for robust levels of both sg mRNAs. Additional analysis of these mutants for minus-strand viral RNA accumulation indicated that the interaction is required at an early step in genome and sg mRNA production, because the disruptions inhibited minus-strand synthesis for both of these classes of viral RNA (Figure 3B, lower panel). The findings with TBSV were confirmed by carrying out the same compensatory mutational analysis in the UL–DL interaction in another tombusvirus, Carnation Italian ringspot virus (CIRV) (Figure 3C). It was also noted, for both viruses, that the dD mutants containing two central UG wobble pairs were ∼three- to fourfold more active than the corresponding dU mutants with CA mismatches (Figure 3B, C, upper panels). This difference suggests that the UL–DL interactions are functionally relevant in the plus-strands of these viral genomes (refer to Figure 3A legend for additional information).

### The UL–DL interaction does not facilitate translation of viral proteins

**Figure 4:**
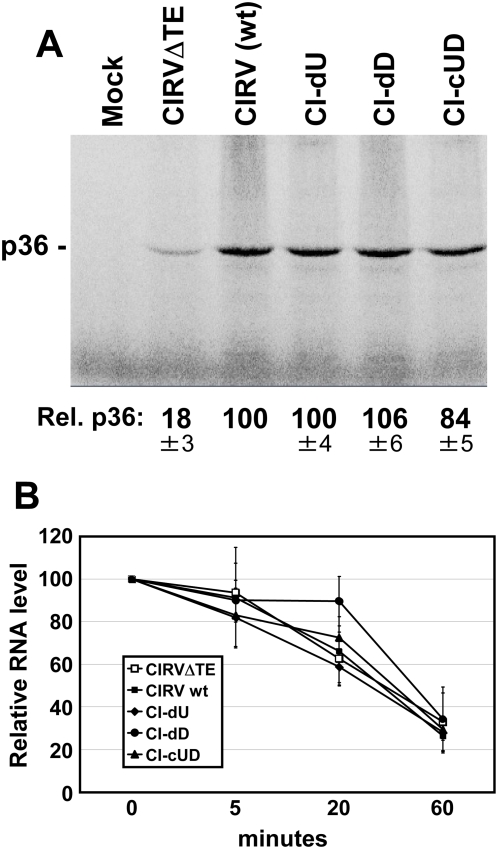
Translational analysis of the CIRV genome in wheat germ extract. (A) SDS-PAGE analysis of proteins synthesized from the CIRV genome and its mutant counterparts. The viral genomes analyzed are labeled above the lanes. The mock lane consists of a translation reaction with no RNA added, while the CIRVΔTE lane contains a genomic control that does not contain a 3′CITE. The position of the expected p36 product is indicated to the left. Products were generated by translating 0.5 pmol of uncapped full-length viral genomes in wheat germ extract for a period of 1 hr at 25°C. The relative accumulation levels of p36 were quantified and the values correspond to means from three independent experiments that were normalized to the accumulation level for the wt genome, set at 100. (B) Stability assay of CIRV genomes in wheat germ extract. Aliquots were removed from translation reactions at various time intervals and the viral RNAs were analyzed by northern blotting. Means of RNA levels (±SD) from three independent experiments were plotted versus time.

The above results indicated a defect at an early step in viral RNA synthesis. One possible explanation for this is that the UL–DL interaction acts to facilitate translation of the RNA replication proteins p33 and p92 from the viral genome. Inhibition of this function would lead to lower levels of viral RNA replication and reduced RNA genome accumulation. Notably, the UL–DL interaction is positioned directly adjacent to the 3′CITE, a location that could potentially aid it in regulating 3′CITE activity (Figure 2A). To address this possibility, the same set of genomic mutants that was analyzed in Figure 3C was assessed in a wheat germ translation extract. CIRV was used for this analysis as, unlike TBSV, its 3′CITE is fully active in this plant-derived in vitro system [31], as illustrated by the significant decrease in translation observed in its absence (*i.e.* mutant CIRVΔTE in Figure 4A). In the translation assay, the level of p36, the homologue of p33 in TBSV, was monitored for the wt CIRV genome and each of its mutants. In general, the viral genomes yielded similar levels of p36, suggesting roughly equivalent efficiencies of translation (Figure 4A). This conclusion was supported by stability analysis of these messages, which showed comparable profiles of RNA decay (Figure 4B). Taken together, these data indicate that the UL–DL interaction does not markedly affect translation of viral proteins.

### The RNA helix formed by the UL–DL interaction is not functionally important

**Figure 5:**
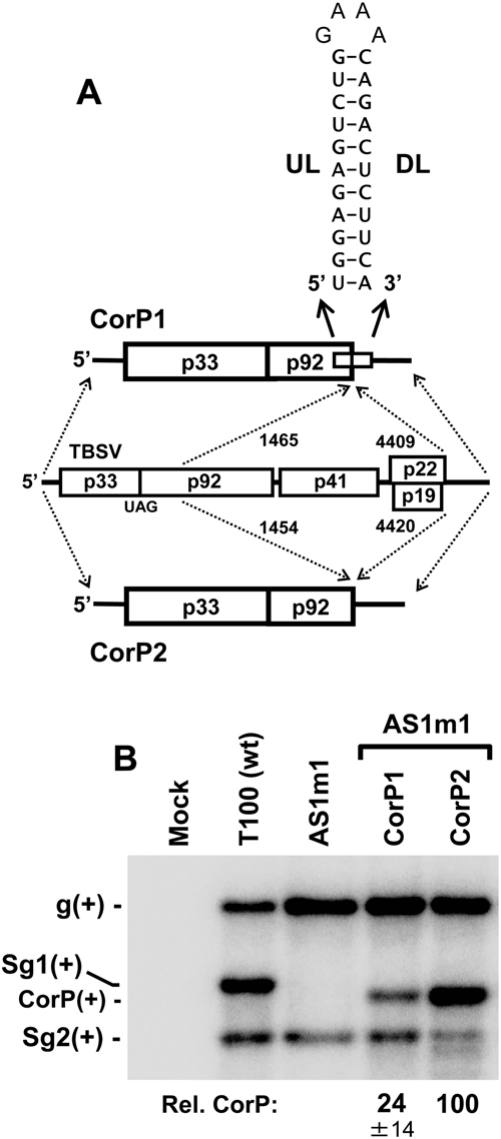
Analysis of the requirement of the UL–DL helix for viral RNA accumulation. (A) Depiction of TBSV-based replicons with deleted intervening sequences that either did (CorP1) or did not (CorP2) include the UL and DL segments. The regions of the TBSV genome (middle) present in CorP1 and CorP2 are shown by the dashed arrows along with corresponding genomic coordinates. The UL–DL sequences, present only in CorP1, are predicted to form the RNA hairpin shown. (B) Northern blot analysis and quantification of CorP replicon accumulation in co-transfections of plant protoplasts.

The lack of involvement of the UL–DL interaction in translation prompted us to refocus our attention to its possible role in directly mediating viral RNA replication. As proposed earlier, the interaction could function simply to juxtapose RII and RIV. However, the potential exists that the RNA helix formed by the UL–DL interaction also contributes to the activity. To investigate this possibility, two mutant TBSV genomes were generated. The first mutant, CorP1, contained the UL and DL sequences, but lacked the intervening sequence between these two partner segments (Figure 5A). To facilitate formation of the UL–DL interaction, the intervening sequence deleted in CorP1 was replaced by a stable GNRA-type tetraloop. In the second mutant, CorP2, the UL and DL sequences, along with their intervening sequence, were removed (Figure 5A). Both CorP mutants were defective for autonomous replication, because they encoded C-terminally truncated p92 ORFs. Consequently, replication of these viral RNAs had to be complemented with a genomic RNA, AS1m1, which provided full-length p92 in trans. AS1m1 does not transcribe sg mRNA1, due to a mutation in its RNA–based transcriptional signal [15], and it was used as the helper genome so as to avoid obscuring the detection of the CorP RNAs, which have lengths that are similar to that of sg mRNA1. The results from co-transfection of AS1m1 with CorP1 into plant protoplasts revealed readily detectable accumulation of the latter; however, the corresponding co-transfection containing CorP2 resulted in significantly higher levels of accumulation of the CorP2 replicon (Figure 5B). The efficient accumulation of the viral replicon lacking the UL–DL interaction indicates that the RNA helix formed by the interaction is not necessary for function and may in fact be somewhat inhibitory to efficient viral RNA replication (though this effect could be related to its stable hairpin context in CorP1). Regardless, these results support the notion that the primary role of the interaction is to mediate the juxtaposition of RII and RIV.

### The UL–DL interaction is important for DI-73 replication but does not directly affect minus-strand synthesis or RNA stability

**Figure 6:**
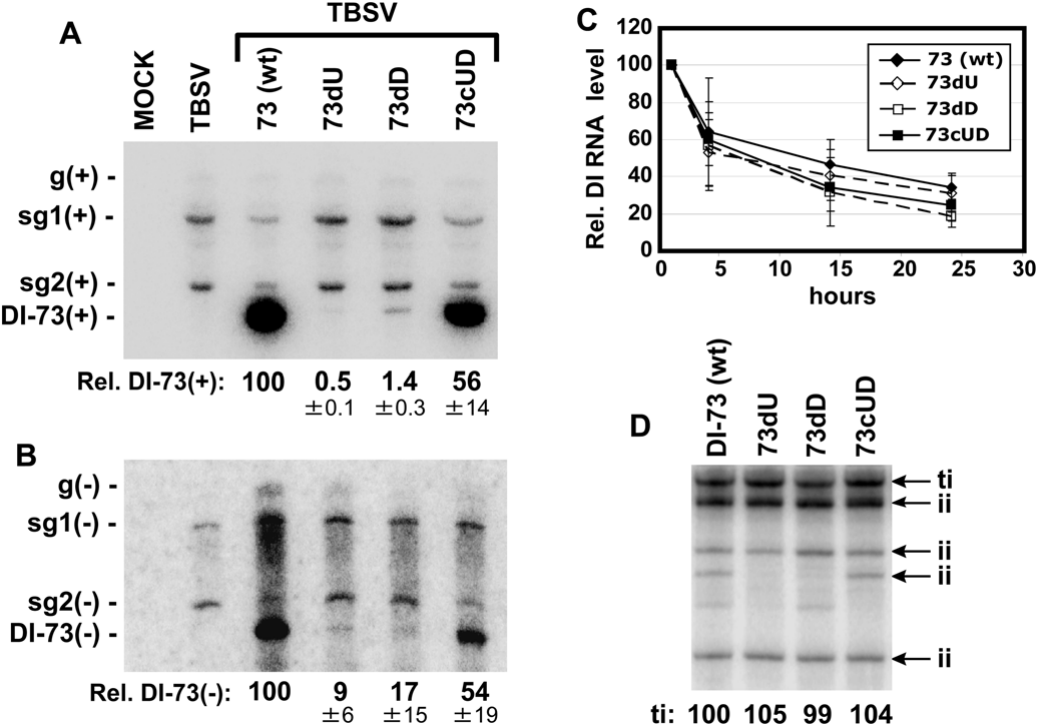
Analysis of the UL–DL interaction in the TBSV DI-73 replicon. (A) Northern blot analysis of the accumulation of DI-73 plus-strands in co-transfections with TBSV genome in plant protoplasts. DI-73 and its variants contained the same set of UL–DL modifications that are shown in Figure 3A. (B) Northern blot analysis of DI-73 minus-strand accumulation. (C) Stability analysis of DI-73 and its mutants in plant protoplasts. Protoplasts were transfected with DI-73 or its variants in the absence of helper genome and the relative levels of these RNAs were determined over time by northern blot analysis. (D) Representative analysis of wt and mutant DI-73 template activities determined in vitro using a plant-derived replicase extract. Terminally-initiated (ti) and internally-initiated (ii) minus-strand products are indicated and levels of the former were quantified.

To further investigate the role of the UL–DL interaction in viral RNA accumulation, we employed the use of a small, non-coding, efficiently replicating, TBSV-derived RNA replicon that requires co-infection with helper genome for its replication [10]. Replicon DI-73 was selected for these studies as it contains both RII and a contiguous 3′ end containing RIII-R3.5-RIV, thereby providing a potentially suitable context to study the UL–DL interaction (Figure 1B) [32]. To validate that the UL–DL interaction was functionally important for its accumulation, the same set of compensatory mutations that was tested in the TBSV genome (Figure 3A) was introduced into DI-73. As observed for the full-length TBSV genome, the disruptive mutations (mutants 73dU and 73dD) led to substantially reduced DI-73 levels of accumulation in plant protoplast co-transfections with helper genome, while restoration of the interaction (mutant 73cUD) led to notably recovered levels of accumulation, both at the plus- and minus-strand levels (Figure 6A and 6B). These results show that even though the UL and DL in DI-73 are separated by only 70 nts, the formation of the interaction is still required. This indicates that the UL–DL interaction is needed to mediate a specific RNA arrangement that is required for optimal function. To determine if the differences in replicon accumulation were related to changes in RNA stability, stability assays were carried out (Figure 6C). No major differences in RNA decay rates were observed, suggesting that the UL–DL-related defect was related to RNA replication efficiency.

The analyses of both genomic and DI-73 replicon RNA accumulation indicated that the UL–DL-based defect in replication affects minus-strand synthesis (Figure 3B and Figure 6B). One possible explanation is that the interaction facilitates folding of the plus-strand viral RNA templates into conformations that are suitable for minus-strand synthesis. To test this idea, DI-73 replicons harboring the compensatory mutations in the interaction were tested in vitro for their ability to serve as templates for minus-strand synthesis using a tombusvirus replicase extract derived from plants [17]. All of the RNA templates tested directed the synthesis of terminally-initiated (ti) minus strands with similar efficiencies (Figure 6D). Internally-initiated (ii) products, commonly observed in such in vitro systems, were, for the most part, also produced at similar levels. These results indicate that the UL–DL interaction neither promotes more efficient minus-strand synthesis by assembled replicase nor acts to redirect internal initiation sites to the 3′-terminus. Accordingly, the UL–DL-related function may act at a step in the RNA replication pathway that precedes minus-strand synthesis.

### The UL–DL interaction facilitates viral replicase assembly

**Figure 7:**
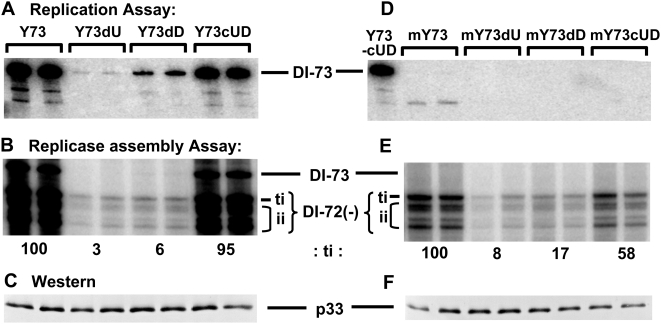
Replication and replicase assembly assays of DI-73 and its mutants in yeast. (A) Representative replication assay showing the accumulation levels of DI-73 (Y73, being the wt yeast plasmid counterpart) and its variants in yeast cells, as assessed by northern blot analysis. These replicons contained the same set of modifications that are shown in Figure 3A. (B) Representative replicase assembly assay showing the efficiency with which affinity-purified replicase (prepared from cells expressing the different DI-73 variants described above) copies an added DI-72(−) template in vitro. Terminally-initiated (ti) and internally-initiated (ii) products are indicated and the former was quantified. (C) Western blot showing levels of p33 present in the cells used for replicase preparation. Similar results were obtained when p92 levels were assessed (not shown). (D, E, F) are as described for (A, B, C), respectively, except that replication-defective forms of DI-73 (designated by the prefix “m”) were used in these assays.

A possible early step in viral RNA replication that could require the UL–DL interaction is replicase assembly. This concept relates back to our original hypothesis that RII and RIV, the only two discontinuous RNA regions required for this process, need to communicate in order to function. Importantly, the juxtaposition of RII and RIV by the UL–DL interaction would satisfy this proposed condition. To address this possibility, a well-defined tombusvirus replicase assembly assay utilizing a yeast-based system was employed [26]. Before carrying out the replicase assembly assay, the DI-73-based compensatory mutants were tested in an in vivo replication assay to determine if the results observed in plant cells could be accurately recapitulated in yeast cells [33]. This replication assay involved expressing p33, p92, and the DI-73 replicon transcript from cotransformed plasmids and monitoring the accumulation of the RNA replicon by northern blot analysis. The relative levels of accumulation of the different viral replicons in yeast were similar to those observed in plant protoplasts, thereby validating use of the yeast system for further investigation (Figure 7A). For the assembly assay, replicase was purified from yeast cells (transformed as described above) and then assayed for its ability to synthesize a complementary strand to an exogenously added viral RNA template, DI-72(−). DI-72(−) is a negative-sense viral RNA and does not contain the UL–DL interaction. Results from this assay showed that the added DI-72(−) RNA template was copied efficiently only in extracts that were isolated from cells containing DI-73 replicons that retained a functional UL–DL interaction (*i.e.* Y73 and Y73cUD; Figure 7B). Western blot analysis indicated that equal levels of p33 were expressed in the cells used for replicase purification (Figure 7C). Next, as a more stringent test of assembly, the wt and compensatory set of DI-73 mutants were modified at their 5′ ends so as to make them replication-defective; as verified by the replication assay (Figure 7D). Under these conditions, DI-73 levels would be similarly low for both wt and mutant forms, thus reducing the effect of replicon levels on the efficiency of replicase assembly. When the replicase assembly assay was carried out under these more strict conditions, a similar trend was observed, where assembly was more efficient when the UL–DL interaction was predicted to be stable (Figure 7E). Collectively, these data indicate that the UL–DL interaction contributes to the efficiency of replicase assembly.

## Discussion

### Long-range RNA–RNA interactions mediate RNA replication in plus-strand RNA viruses

**Figure 8:**
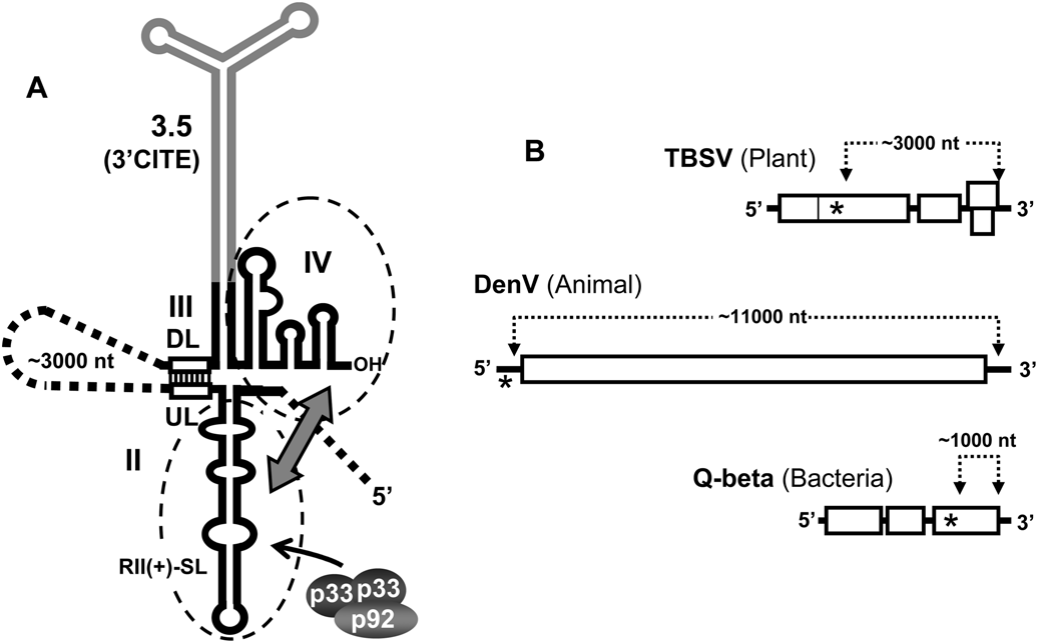
Secondary structures of portions of the TBSV genome depicting the UL–DL interaction and linear representations of similar interactions in other viruses. (A) Secondary structure cartoon showing RII and RIII-R3.5-RIV and the UL–DL interaction (open rectangles). The RNA elements that are essential for replicase assembly are enclosed by dashed ovals, and the double-headed arrow depicts the communication required between these structures. p33 and p92 are shown as shaded ovals and the arrow indicates binding to RII(+)-SL. (B) Linear representation of positive-strand RNA viral genomes that infect hosts from three different kingdoms. The long-range RNA–RNA interactions in these viral genomes that are required for RNA replication are depicted by dotted lines, with the approximate lengths of the intervening sequences indicated. The approximate positions of replicase binding sites are indicated by asterisks.

We have identified a novel long-distance RNA–RNA interaction in the TBSV genome that mediates viral RNA replication by facilitating viral replicase assembly. Our results are consistent with the recognized critical roles for RII and RIV in this process [26] and they indicate that the mere presence of both of these regions in the genome is not sufficient for optimal function. Based on our findings, we propose a model in which efficient formation of the viral RNA replicase requires RII and RIV to be spatially united at some point during the assembly process (Figure 8A).

Different mechanistic variants can be envisioned for how the UL–DL interaction facilitates assembly of the replicase by forming a discontinuous RNA platform. The three schemes presented here, however, are neither exhaustive nor necessarily mutually exclusive; moreover, hybrid versions are also possible. Scheme 1: The UL–DL interaction forms first and allows proteins to associate with either RII or RIV; protein-protein interactions then mediate replicase assembly. Scheme 2: The juxtaposed RII and RIV together form a discontinuous binding site that recruits an important component(s) necessary for replicase assembly. Scheme 3: Protein factors bind to the individual non-united RII and RIV elements; the UL–DL interaction then juxtaposes the bound factors, which mediates replicase assembly. With respect to the latter scheme, studies have shown that viral p33 is able to bind in vitro to RII(+)-SL in the absence of other RNA elements [24]. This suggests that, for at least some factors, formation of the UL–DL interaction may not need to precede protein binding. Also, as p92 associates with RII by interacting with p33 [24],[25] (Figure 8A), a secondary function for the UL–DL interaction may be to reposition the internally-bound p92 RdRp close to the 3′ terminus of the RNA genome, thereby allowing it to efficiently initiate minus-strand synthesis (Figure 8A). This function, however, does not seem necessary for assembled replicase, as such complexes were able to effectively initiate minus-strand synthesis in vitro irrespective of the UL–DL interaction and the interaction did not facilitate more efficient terminal initiation (Figure 6D). Nonetheless, if replicase assembly is tightly coupled to minus-strand synthesis in vivo, it is possible that the UL–DL interaction also mediates 3′-end positioning of the p92 RdRp. Indeed, this type of repositioning mechanism has been implicated in facilitating the function of an RNA hairpin enhancer element present in the minus-strand of a small TBSV defective interfering RNA [34].

Interestingly, RdRp-positioning roles for long-distance RNA–RNA interactions have also been proposed for two different plus-strand RNA viruses that infect animal and bacterial hosts. For Dengue virus (DenV), an RNA–based interaction spanning ∼11,000 nts in its genome has been proposed to reposition the viral RdRp, bound to its 5′-proximal promoter, close to the 3′-end of the genome; thereby allowing it to efficiently initiate minus-strand synthesis [35] (Figure 8B). Similarly for Q-beta bacteriophage, the viral replicase was shown to bind to an internal site in the RNA genome that is juxtaposed to the 3′-terminus by an RNA-mediated interaction spanning ∼1000 nts [36] (Figure 8B). Long-range genomic RNA–RNA interactions involved in viral RNA replication are thus prevalent and include viruses that infect organisms from three different kingdoms (Figure 8B). Interestingly, all three of these viruses belong to the supergroup-II class of RdRps [37], which suggests that in addition to having a common polymerase ancestry, they also share a history of employing long-distance RNA–RNA interactions in their RNA replication processes. Possible selective advantages for separating codependent RNA replication elements throughout a viral genome have been reported [35], *e.g.* preferentially facilitating the amplification of full-length RNA templates, and such advantages may also be relevant to TBSV. However, TBSV is distinct from both DenV and Q-beta phage in that it produces 3′-coterminal sg mRNAs (Figure 1A). Notably, these sg mRNAs do not include RII in their structure, a feature that would preclude replicase formation on these messages and could act to prevent interference with their primary function of viral protein translation. Accordingly, the underlying principles behind spatially separating functional RNA elements are likely varied and are undoubtedly influenced by viral reproductive strategy.

### An integrated model for global viral genome structure

**Figure 9:**
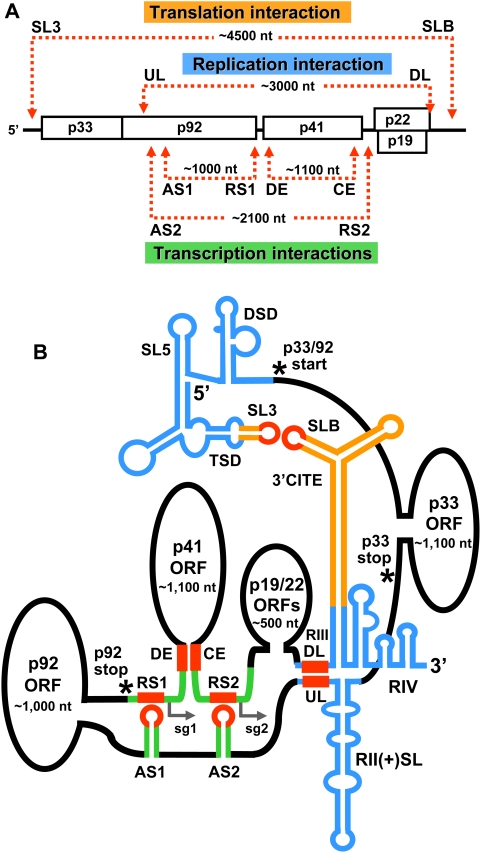
Linear and higher-order structural models for the functional long-distance RNA–RNA interactions that occur in the TBSV genome. (A) Linear representation of the TBSV genome showing RNA–based interactions involved in translation, replication, and sg mRNA transcription. (B) Higher-order structural model for long-range RNA–based interactions in the TBSV genome. The sequences that are directly involved in forming long-range base pairing interactions are shown in red, while associated sequences and/or structures that are involved in translation, replication, and sg mRNA transcription are color coded as orange, blue, and green, respectively. Relevant structures are labeled (TSD, T-shaped domain; DSD, downstream domain; AS, activator sequence; RS, receptor sequence; CE, core element; DE, distal element). The SL3–SLB interaction is required for translation of p33/92 from the genome. The AS1–RS1 interaction is required for sg mRNA1 transcription, while both the AS2–RS2 and DE–CE interactions are required for sg mRNA2 transcription. The TSD, SL5, and DSD are located 5′-proximally in the 5′UTR of the genome and are involved in mediating genome plus-strand RNA synthesis [65]. Large intervening sections of sequence, which are predicted by mfold to form domains (see Figure S1), are shown as ovals and roughly correspond to the ORFs for p33, p92, p41, and p19/22 (not to scale). The start codon for p33/92, as well as the termination codons for these two proteins, are labeled and denoted by asterisks. Initiation sites of the two sg mRNAs are indicated by small arrows. See text for additional details.

The discovery of a long-range RNA–RNA interaction in the TBSV genome that is involved in RNA replication is significant, because it designates this virus as the first shown to use distal RNA–based communication in three different fundamental viral processes, the other two being cap-independent translation and sg mRNA transcription (Figure 9A). Importantly, this finding has allowed us to generate a comprehensive higher-order RNA structural model of functional long-range interactions in the genome of this eukaryotic RNA virus (Figure 9B). This model effectively illustrates that the different interactions are highly integrated and thus require a significant level of coordination in order to function properly (Figure 9B). Indeed, the newly identified UL–DL interaction places replication elements RII and RIV close to both translational (*i.e.* 3′CITE/5′UTR) and transcriptional (*i.e.* AS1/RS1 and AS2/RS2) RNA elements (Figure 9B). Thus, if all of the long-range interactions were to occur at once, as is depicted in Figure 9B, they would form a core of regulatory RNA elements. Notably, the large intervening segments, which are primarily coding regions, are predicted by mfold to form discrete domains (Figure S1). Based on this proposed configuration, the different regulatory RNA elements (and any associated proteins) could potentially communicate directly with each other in a common zone that could be used for coordinating different viral processes and/or sharing protein factors (Figure 9B).

Temporally, initiation of a viral infection would start with translation, followed by replication (*i.e.* replicase assembly), and then transcription. Accordingly, each of the different types of functional long-range RNA–based interactions would need to be dynamic and able to form in parallel with this series of events. Mechanistically, some of these interactions are predicted to be inhibitory to others and, accordingly, some processes may be impeded by others. Such relationships could be integral to coordinating different events or may simply act as safe-guards to prevent two processes from occurring simultaneously. For example, the UL–DL interaction would be inhibited by translation that is mediated by the 5′-3′ SL3-SLB interaction, because ribosomes translating the read-through portion of the p92 ORF would disrupt the UL–DL interaction (as well as the RII structure) (Figure 9B). Conversely, minus-strand synthesis of the genome, which is mediated by the UL–DL interaction, would cause the actively copying RdRp to disrupt the translation-related SL3-SLB interaction (as well as the 3′CITE structure), thereby inhibiting translation (Figure 9B), as has been proposed for Barley yellow dwarf virus (BYDV) [38]. Such incompatibilities could serve regulatory functions and aid the virus in switching from one process to another (*e.g.* translation to replication; [38]). In other cases, interactions, such as the UL–DL replication-related interaction and the AS-RS transcription-related interactions, may be mechanistically compatible, because both of the associated viral processes require the RdRp and initiation of minus-strand synthesis [39]. The higher-order RNA genome structural model in Figure 9 serves to illustrate an extraordinary level of integration of long-range RNA–based contacts and extends our fundamental view of RNA virus genome structure and function. Importantly, it also provides a useful molecular framework for future studies aimed at unraveling the dynamic regulatory interplay between these diverse sets of interactions.

### Prevalence of long-range riboregulation in RNA viruses

TBSV represents an extreme example of long-range RNA–RNA interactions participating in three distinct viral processes. Similar types of distal RNA networks have also been reported in other positive-strand RNA viruses. For instance, the luteovirus BYDV utilizes two distinct sets of long-range RNA–based interactions for mediating translational initiation [40] and readthrough [38]. The requirement for base pairing between terminal genomic UTRs for efficient translation was shown initially in BYDV [40] and subsequently in TBSV [12],[13]. Interestingly, a recent report on the nepovirus Blackcurrant reversion virus distinguishes it as an additional virus confirmed to have this terminal pairing requirement [41]. In contrast, translational regulation in the bacteriophage MS2 involves internally located RNA–based interactions that modulate internal initiation events [42]. Accordingly, both prokaryotic and eukaryotic ribosome function can be modulated by the activity of this type of RNA interaction.

Sg mRNA transcription in the nodavirus Flock house virus involves an intra-genomic long-distance interaction [43] that likely functions in a manner similar to those that mediate transcription in TBSV [14]–[16]. The dianthovirus Red clover necrotic mosaic virus is also proposed to use an analogous premature termination mechanism; however it represents an extraordinary case in which transcription requires interaction between its two genomic RNA segments [44]. More recently, a distal intra-genomic interaction in the coronavirus Transmissible gastroenteritis virus was discovered that acts as an enhancer of sg mRNA transcription [8]. Collectively, these findings indicate both primary and secondary roles for these interactions in the process of sg mRNA transcription.

For genome replication, the potexvirus Potato virus X (PVX) utilizes an extensive set of long-range RNA–RNA interactions [6] that, interestingly, are also involved in sg mRNA transcription [45]. Unlike the viruses presented in Figure 8B, PVX possesses a supergroup-III RdRp [37]. Additionally, its functional interactions differ from those described for the supergroup-II RdRp viruses in that the primary sequence of the participating RNA segments, in addition to their complementarity, is important for activity [6],[45]. This extra requirement suggests a mechanism that is more complex than the bridging functions proposed for TBSV, DenV and Q-beta phage.

A role for long-range intra-genomic interactions in viral replication has also been demonstrated for different flaviviruses [9], [46]–[50], as well as for related Hepatitis C virus [7], [51]–[53]. Moreover, important long-distance RNA–RNA interactions have been described for both minus-strand RNA viruses [54]–[57] and retroviruses [58],[59]. This ever growing list of diverse RNA viruses illustrates both the prevalence and fundamental importance of long-range RNA–RNA interactions in a wide assortment of reproductive strategies.

Conceptualizing viral RNA genomes as complex higher-order RNA structures will provide valuable models for understanding their many functions. Indeed, recent computationally-based structural studies suggest that viral genome-scale ordered RNA structures (GORS) are more prevalent than previously appreciated [60],[61] and atomic force microscopic analysis has shown that viral RNA genomes are capable of adopting pseudo-globular conformations [61]. These findings further underscore the importance of considering overall RNA structure when investigating the roles of viral RNA genomes. Indeed, increasing our knowledge of global RNA structure and function will improve our understanding of mechanistic features of virus reproduction, facilitate the engineering of effective viral vectors, and help define genome-level structural constraints that influence RNA virus genome evolution.

## Methods

### Plasmid construction

Constructs described previously that were used in plant-based experiments include: T100, the wt TBSV genome construct [11]; AS1m1, a modified TBSV genome with sg mRNA1 transcription inactivated [15] and; DI-73, a small TBSV-based replicon [32]. Using the above constructs, and the infectious clone of CIRV [62], additional viral mutants were made and the relevant modifications are presented in the accompanying figures. For yeast-based experiments, the p33- and p92-expression constructs pGBK-His33 and pGAD-His92 have been described previously [33]. The newly generated DI-73 containing plasmids (Y73 series; Figure 7) were based on pYES-DI-72(+)Rz [33] and expressed full-length DI-73(+) RNA transcripts in yeast cells. The corresponding replication-defective mY73 series contained substitutions in RI that are described elsewhere (see mutant m2 in Figure 6 in [63]). All modifications were made using PCR-based mutagenesis and standard cloning techniques [64]. The PCR-derived regions in the constructs were sequenced completely to ensure that only the intended modifications were present.

### In vitro transcription, protoplast transfection, and viral RNA analysis

In vitro RNA transcripts of viral RNAs were generated using T7 RNA polymerase as described previously [32]. Preparation and transfection of cucumber protoplasts and extraction of total nucleic acids were carried out as outlined in Choi and White, 2002 [15]. Briefly, isolated cucumber protoplasts (∼300,000) were transfected with RNA transcripts (3 µg for genomic RNA; 1 µg for replicon RNA) and incubated at 22°C for 22 hr. Isolated total nucleic acid preparations were subjected to northern blot analysis to detect plus- and minus-strand viral RNAs as described previously [15]. Nucleic acids were either treated with glyoxal and separated in 1.4% agarose gels or denatured in formamide-containing buffer and separated in 4.5% polyacrylamide-8% urea gels. Equal loading for all samples was confirmed prior to transfer via staining the gels with ethidium bromide. Viral RNAs were detected using strand-specific ^32^P-labeled probes and relative isotopic levels were determined using PharosFx Plus Molecular Imager (BioRad).

DI RNA stability assays were performed as described previously [63] and RNA secondary structures were predicted using mfold version 3.2 [29],[30].

### Replicase assays

Plant-derived replicase assays were carried out as described previously [17]. Briefly, extracts prepared from tombusvirus-infected *N. benthamiana* plants were supplemented with a buffer containing NTPs (UTP being labeled), viral RNA templates and other components. The reactions were then incubated at 25°C for 120 min. After phenol/chloroform extraction and ammonium acetate/isopropanol precipitation, half the amount of the RNA products was analyzed in 5% polyacrylamide-8% urea gels followed by detection by autoradiography.

Yeast replication assays were carried out in whole cells expressing p33, p92, and the RNA replicon transcript from cotransformed plasmids, as described previously [33]. Following induction and an incubation period, isolated viral RNAs were separated in 5% polyacrylamide-8% urea gels and viral RNA was detected by northern blot analysis. For yeast replicase assembly assays, viral replicase was prepared from yeast cells (transformed as described above) by affinity purification as described elsewhere [26]. The level of replicase activity was determined by adding minus-strand DI-72, DI-72(-) and assessing the amount of ^32^P-labeled complementary-strand product generated by 5% polyacrylamide-8% urea gel electrophoresis and autoradiography. Standard western blot analysis was used to monitor the levels of p33 expressed in the cells used for replicase purification [26].

### In vitro translation

Translation of sub-saturating amounts (0.5 pmol) of RNA transcript was carried out in nuclease-treated wheat germ extract (Promega) as described previously [31]. Protein products were separated by sodium dodecyl sulfate-polyacrylamide gel electrophoresis and quantified by radioanalytical scanning using a PharosFx Plus Molecular Imager (Bio-Rad) and QuantityOne software (Bio-Rad).

## Supporting Information

Figure S1 Optimal RNA secondary structure predicted for full-length TBSV RNA genome as determined by mfold analysis.
(1.91 MB PDF)
