# Supplementary material for: A Discontinuous RNA Platform Mediates RNA Virus Replication: Building an Integrated Model for RNA–based Regulation of Viral Processes
Source: PLoS Pathog. 2009 Mar 6;5(3):e1000323. doi: 10.1371/journal.ppat.1000323 (PMC2648310; doi:10.1371/journal.ppat.1000323)
Supplement: Figure S1 — Optimal RNA secondary structure predicted for full-length TBSV RNA genome as determined by mfold analysis. (1.91 MB PDF) [file ppat.1000323.s001.pdf]

Figure S1: Optimal RNA secondary structure predicted for full-length TBSV RNA genome as determined by mfold analysis. RNA folding analyses were performed using default settings of mfold version 3.2 and no constraints (*i.e.* forced pairings) were imposed. Large sections of intervening RNA, which were predicted to form distinct domains, are denoted with ovals and labeled according to the viral coding sequence that they include (cf. with Figure 9B). A similar overall genome structure and domain organization was also predicted by mfold for twelve other sequenced species in the tombusvirus genus (not shown). RNA elements involved in functional long-distance interactions in the TBSV structure are shown and relevant viral regions (RI, RII, RIII, R3.5 and RIV) are labeled. In the mfold-predicted structure, RI and RIV interact directly, however both regions have been shown experimentally to form functionally-relevant localized RNA structures – as depicted in Figure 9B. Moreover, both RI and RIV contain intra-regional tertiary RNA interactions (*i.e*. pseudoknots), not presented in Figure 9B, that stabilize them. Mfolds analysis does not detect pseudoknots or include their free energy contributions into structure calculations; therefore, the alternative long-range pairing shown in the mfold-predicted structure is likely a consequence of this limitation. Nonetheless, mfold did correctly predict and include several known long-distance interactions that are functionally relevant: (i) the UL-DL interaction, (ii) the DE-CE interaction, and (iii) the AS1-RS1 interaction. Notably, the AS2 and RS2 elements, which form a fourth functional interaction, are in close proximity in the predicted structure. Similarly, the components of the fifth functional interaction, between SL3 in RI and SLB in R3.5 (3'CITE), are also in close relative proximity in the folded structure. Overall, this predicted structure (ΔG = -1588), which includes several large domains, is consistent with the formation of all functional long-range RNA-RNA interactions currently known to occur in the TBSV genome (as depicted in Figure 9B).

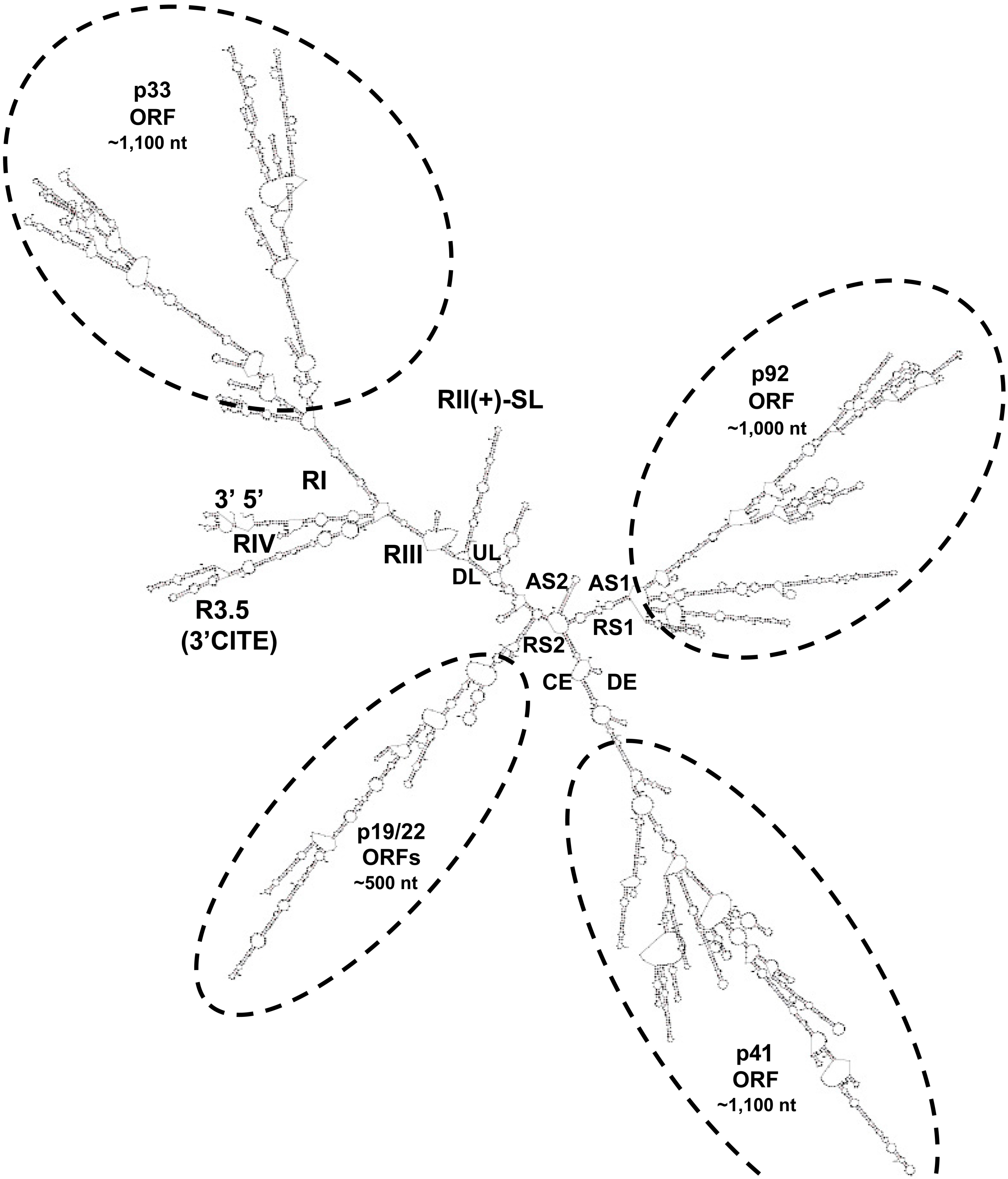
p33
ORF
~1,100 nt
RII(+)-SL
p92
ORF
~1,000 nt
RI
3' 5'
RIV
RIII
UL
DL
AS2
AS1
R3.5
(3'CITE)
RS1
RS2
CE
DE
p19/22
ORFs
~500 nt
p41
ORF
~1,100 nt
